# Atypical Clinical Manifestations of Graves' Disease: An Analysis in Depth

**DOI:** 10.1155/2012/768019

**Published:** 2011-11-01

**Authors:** Mohamed Osama Hegazi, Sherif Ahmed

**Affiliations:** Medical Department, Al Adan Hospital, P.O. Box 262, Hadiya 52853, Kuwait

## Abstract

Over the past few decades, there has been an increase in the number of reports about newly recognized (atypical or unusual) manifestations of Graves' disease (GD), that are related to various body systems. One of these manifestations is sometimes the main presenting feature of GD. Some of the atypical manifestations are specifically related to GD, while others are also similarly seen in patients with other forms of hyperthyroidism. Lack of knowledge of the association between these findings and GD may lead to delay in diagnosis, misdiagnosis, or unnecessary investigations. The atypical clinical presentations of GD include anemia, vomiting, jaundice, and right heart failure. There is one type of anemia that is not explained by any of the known etiological factors and responds well to hyperthyroidism treatment. This type of anemia resembles anemia of chronic disease and may be termed GD anemia. Other forms of anemia that are associated with GD include pernicious anemia, iron deficiency anemia of celiac disease, and autoimmune hemolytic anemia. Vomiting has been reported as a presenting feature of Graves' disease. Some cases had the typical findings of hyperthyroidism initially masked, and the vomiting did not improve until hyperthyroidism has been detected and treated. Hyperthyroidism may present with jaundice, and on the other hand, deep jaundice may develop with the onset of overt hyperthyroidism in previously compensated chronic liver disease patients. Pulmonary hypertension is reported to be associated with GD and to respond to its treatment. GD-related pulmonary hypertension may be so severe to produce isolated right-sided heart failure that is occasionally found as the presenting manifestation of GD.

## 1. Introduction

Graves' disease (GD) accounts for up to 80% of hyperthyroidism cases and is estimated to affect 0.5% of the population [1]. It usually presents with the common well- known symptoms and signs (goiter, ophthalmopathy, weight loss, nervousness, tremors, palpitations, sweating, etc.) which are the distinctive features of the disease (Table 1). We can observe another group of manifestations, such as periodic paralysis, apathy, or psychosis, which are less common and less distinctive despite being well documented in relation to GD (Table 1). Over the past few decades, there has been an increase in the number of reports about newly recognized (atypical or unusual) manifestations of hyperthyroidism that are related to various body systems and may create a wide range of differential diagnosis [2, 3]. Most of these atypical manifestations are mainly reported in patients with GD (Table 1), either due to a specific relation to the autoimmune thyroid disorder, or because GD accounts for the majority of hyperthyroidism cases. Occasionally, one of the atypical manifestations is the main presenting feature of GD [2]. Lack of knowledge of the association between these findings and GD may lead to delay in diagnosis, misdiagnosis, or unnecessary investigations. 

The atypical manifestations of GD represent a wide spectrum of clinical and laboratory findings, and in this review we will focus on the clinical part of that spectrum. For example, while hematological manifestations of GD include thrombocytopenia, leucopenia, anemia, and pancytopenia; we will discuss anemia as the clinical presenting feature. Other atypical clinical presentations of GD that will be discussed here are vomiting, jaundice, and right heart failure. These manifestations can be attributed to a wide variety of hematological, gastrointestinal, and cardiopulmonary causes, and each of them represents a very common clinical condition.

## 2. Anemia

Anemia is not uncommonly found in association with GD. It has been found in 33% of GD patients [4], and was a presenting manifestation in up to 34% of cases with hyperthyroidism [5]. It is somewhat challenging to face anemia as the presenting manifestation of GD, especially when the typical clinical features of hyperthyroidism are subtle or overlooked. Regardless of the incidental association of GD with other forms of anemia (e.g., iron deficiency anemia, thalassemia, etc.), there are specific types of anemia that are directly or indirectly related to GD (Table 2). As an autoimmune disease, GD was found to be associated with other autoimmune diseases that include pernicious anemia, celiac disease, and autoimmune hemolytic anemia [6, 7]. Moreover, there is a certain type of anemia that occurs with Graves' disease and remains unexplained after excluding all other possible causes [4, 8]. Because of its clear relation to GD, and its cure following hyperthyroidism treatment, this type of anemia may be termed GD anemia [4].

### 2.1. Graves' Disease Anemia

In the study by Gianoukakis et al., GD anemia was found in 22% of GD patients [4]. In GD anemia the mean corpuscular volume (MCV) could be normal [8] or, probably more commonly, low [4, 9]. Generally the anemia that coexists with GD is observed to be mild and is commoner with severe disease [5] When GD anemia is microcytic, iron indices are normal and hereditary hemoglobinopathies are readily excluded [10]. Anemia may be the sole haematological abnormality, or it may be combined with thrombocytopenia, or leucopenia; and occasionally it may be present as a part of a GD-associated pancytopenia [9, 11, 12]. Erythropoietin levels are within normal reference ranges [4] and bone marrow, if examined, is hypercellular or, less commonly, normocellular; with normal iron stores [9, 13]. The exact pathogenesis of GD anemia remains unclear [8]; however an effect of the excess thyroid hormones has been postulated [10]. The hypercellular marrow may indicate that erythropoiesis is enhanced due to hyperthyroidism, but in the same time it is ineffective, hence the finding of anemia with low MCV [10]. Hematologically, anemia in the presence of hypercellular marrow could be related to either organ sequestration such as observed in hypersplenism, an enhanced removal of circulating red blood cells by an immune or toxic mechanism, or a hemopoietic stem cell dysfunction such as myelodysplasia [9]. One or both of the latter 2 mechanisms could be responsible for the GD anemia, with myelodysplasia being the most widely accepted explanation [9, 10, 13]. The finding that thyroid-stimulating hormone (TSH) receptor antibodies nonspecifically attach to the surface of the red blood cells, may suggest an autoimmune basis for GD anemia [14]. However, the rare occurrence of GD anemia with hyperthyroid nodular goiter (toxic multinodular goiter and toxic adenoma) makes the effect of thyroid hormones on hemopoiesis a more likely explanation than the autoimmune mechanism [12, 13]. Generally, GD anemia resembles anemia of chronic disease in many aspects including red cell morphology, iron status, erythropoietin levels, and association with markers of inflammation [4]. GD anemia was observed to correct promptly with return to the euthyroid state following hyperthyroidism treatment [4, 9, 10, 12, 13]. Correction included normalisation of the haemoglobin concentration and also of the MCV [4, 9, 10]. This improvement was observed regardless of the mode of therapy of hyperthyroidism, with antithyroid drugs being the more commonly used agents in this regard [4, 9, 10, 13].

### 2.2. Pernicious Anemia

Pernicious anemia is a well-known form of the autoimmune diseases that may occur in association with GD [6, 7, 15]. In the study by Boelaert et al., the prevalence of pernicious anemia among patients with GD was 1.4% compared to 0.13% in the UK general population [7]. The finding of megaloblastic anemia (marked macrocytosis with hypersegmanted polymorphonuclear leukocytes) in the peripheral blood film of a GD patient should raise the suspicion of this association. Anemia may be associated with leukopenia or thrombocytopenia; or it could form a part of the pancytopenia of pernicious anemia [16]. The diagnostic workup is a straight forward one and includes checking serum vitamin B12 concentration, red cell or serum folate concentration (to rule out folate deficiency), anti-intrinsic factor antibody gastric parietal cell antibody and the Schilling test.

### 2.3. Iron Deficiency Anemia due to Celiac Disease

 In general, the major cause of iron deficiency anemia (microcytic anemia with a low iron status) is blood loss, either overt or occult [17]. Lack of evidence of blood loss, or the refractoriness to treatment with oral iron may lead to the suspicion of celiac disease. In GD patients, the presence of an iron deficiency anemia may indicate an associated celiac disease, but of course it does not mean omitting blood loss as a common possible cause. In the study by Boelaert et al., the prevalence of celiac disease was 0.9% in GD patients compared to 0.047% in the general UK population [7]. Review of the literature also showed that asymptomatic cases of celiac disease were detected when patients with autoimmune thyroid disease (including GD) were screened by autoantibody testing and duodenal biopsy [18]. However, Sattar et al. stated that screening for celiac disease in patients with autoimmune thyroid disease may not be justified without comorbidities or symptoms [19]. When GD and celiac disease co-exist, it is not clear whether the treatment of one of them affects the course of the other, but it is interesting to mention that treatment with a gluten-free diet has been associated with improvement in the coexistent Hashimoto's hypothyroidism, with reduction of the required thyroxine doses an effect probably related to enhanced drug absorption [18].

### 2.4. Autoimmune Haemolytic Anemia

The association of GD with autoimmune haemolytic anemia has been described in single case reports in the English and non-English literatures [20–23]. It appears that autoimmune haemolytic anemia is much less commonly found in association with GD when compared with immune thrombocytopenia and pernicious anemia [24]. In some of the case reports, autoimmune haemolytic anemia was present as a part of Evans' syndrome (autoimmune haemolytic anemia and idiopathic thrombocytopenic purpura) in association with GD [25, 26]. In the study by Rajic et al., on 362 subjects with autoimmune haematological disorders, there was no evidence of simultaneous autoimmune thyroid disease in the subgroup of patients with autoimmune haemolytic anemia [24]. Ikeda et al. reported a case of Evans' syndrome in a patient with GD that was not hyperthyroid after treatment with radioiodine, and suggested that an underlying immunological mechanism could be responsible for the association [25]. In this regard it was very interesting to get an effective control of hemolysis with the use of an antithyroid drug alone (namely, propylthiouracil) that was observed in a case of autoimmune haemolytic anemia [20], and in another one with Evan's syndrome [26]. This finding might be related to the earlier observation that microsomal antibodies and TSH receptor antibodies decreased in parallel, while patients with GD were taking carbimazole, whereas no significant changes were observed during treatment with placebo or propranolol [27]. The changes in autoantibody levels during carbimazole treatment were independent of changes in serum thyroxine and could have been due to a direct effect of the drug on autoantibody synthesis [27].

## 3. Vomiting

Vomiting is one of the most common symptoms of gastrointestinal disease. Patients with GD may present mainly with gastrointestinal symptoms that include diarrhea, frequent defecation, dyspepsia, nausea, vomiting, and abdominal pain [28]. A special clinical situation arises when a thyrotoxic patient, who lacks the typical unique features of hyperthyroidism, presents with severe and persistent vomiting. In one of the earliest reports, Rosenthal et al. described 7 patients with thyrotoxic vomiting with a delay in the detection of hyperthyroidism of 8 & 17 months in two of the cases [29]. Lack of awareness about the association between vomiting and hyperthyroidism may lead to a more marked delay in the diagnosis; that was 7 years in one case report [30]. In a review of 25 newly diagnosed thyrotoxicosis cases 44% of subjects were complaining of vomiting [31]. The mechanism by which vomiting develops in hyperthyroid patients remains uncertain [32]. Researchers have documented increased levels of estrogens in patients of both sexes with thyrotoxicosis [32]. Estrogens may act as an emetic agent with individual variation in susceptibility between patients [32]. Another postulated mechanism is through an increase in beta adrenergic activity due to an increased number of beta adrenergic receptors in hyperthyroid patients [32]. This mechanism has been concluded from the finding of increased adrenergic activity in hyperthyroidism [33], and from the observation that starting treatment with beta blockers ameliorates the vomiting in some cases [32]. However, such an explanation may be debated, as vomiting is more likely to be linked to hypo-, rather than hyper-adrenalism. In addition, the beneficial effect of beta blockers could be due to the reduced thyroid hormone activity (reduced T3 concentration) and not due to a decrease in beta adrenergic activity. Another possible mechanism is through the effect of excess thyroid hormones on gastric motility. Thyroid hormones are thought to decrease gastric emptying secondary to a malfunction of the pyloric sphincter [32]. In a study on 23 patients with hyperthyroidism, 50% had delayed gastric emptying [34]. In another study, a slight but a statistically significant increase in the rate of gastric emptying occurred in patients after restoration of euthyroidism as compared with healthy control subjects [35]. In almost all reports, thyrotoxic vomiting showed an excellent improvement either within several days after the initiation of antithyroid treatment, or in temporal relation with the return to the euthyroid state [29, 30, 32]. 

### 3.1. Hyperthyroidism with Vomiting in Pregnancy

Vomiting is common in pregnancy and pregnant women are frequently checked for thyroid disorders [36, 37]. Hyperemesis gravidarum (HG) is known to be associated with mild transient hyperthyroidism probably due to the thyroid stimulating effect of human chorionic gondotropin [36–39]. On the other hand, frank hyperthyroidism is not infrequently discovered for the first time during pregnancy with GD being the most common cause [36, 40, 41]. Moreover, hyperthyroidism occurs in pregnancy with clinical presentation similar to HG and pregnancy itself [36, 41].

A common, challenging scenario develops when a pregnant lady gets severe vomiting together with a biochemical evidence of hyperthyroidism. Here she could be having either transient hyperthyroidism that is associated with HG, or overt hyperthyroidism that manifests with vomiting. It is important to differentiate between the two conditions (Table 3) because transient hyperthyroidism with HG is usually mild, self-limited, and requires no treatment [36, 37]; while frank hyperthyroidism (due to GD in 90% of cases) confers high maternal and fetal morbidity and mortality, and needs to be early detected and treated [36, 40, 41]. The presence of marked tachycardia, tremors, muscle weakness, and ophthalmopathy make the diagnosis of frank hyperthyroidism more likely (Table 3). Goiter especially if associated with a thyroid bruit may point to GD, but one should bear in mind that the thyroid gland may physiologically enlarge during normal pregnancy [41]. The presence of severe vomiting makes HG the likely diagnosis only with the exception of the unusual situation when vomiting is the main presenting symptom of thyrotoxicosis. Biochemically, transient hyperthyroidism of HG usually shows a picture of subclinical hyperthyroidism (Low TSH and normal free T4). The diagnosis of overt hyperthyroidism in pregnant women should be based primarily on a serum TSH value <0.01 mU/L and also a high serum-free T4 value [42]. Free T3 measurements may be useful in women with significantly suppressed serum TSH concentrations and normal or minimally elevated free T4 values [42]. Thyroid peroxidase antibodies are markers of autoimmune thyroid disease in general and will not differentiate as they are found in a considerable percentage of pregnant women. TSH receptor antibodies may help to indicate that GD is the cause of the overt hyperthyroidism. Finally, if the clinical and/or the biochemical hyperthyroidism persist beyond the first trimester, causes of hyperthyroidism other than HG should be actively sought, putting in mind that some 10% of women with HG may continue to have symptoms throughout pregnancy [40].

## 4. Jaundice

The spectrum of liver affection in GD extends from asymptomatic biochemical abnormality to frank hepatitis [3, 43]. In the vast majority of cases it is only the biochemical abnormality that attracts the physician rather than the clinically obvious liver disease [3, 43, 44]. Liver function derangement in hyperthyroid patients can be mainly subdivided into either transaminases elevations (hepatocellular pattern), or intrahepatic cholestasis [3, 43, 45]. In a study by Gürlek et al., at least one liver function test abnormality was found in 60.5% of hyperthyroid patients [44]. Elevations of alkaline phosphatase, alanine aminotransferase, and gamma-glutamyl transpeptidase levels were observed in 44%, 23%, and 14% of the patients, respectively [44]. The mechanism of hepatic injury appears to be relative hypoxia in the perivenular regions, due to an increase in hepatic oxygen demand without an appropriate increase in hepatic blood flow [46]. One theory suggests that the liver is damaged by the systemic effects of excess thyroid hormones [47]. The hypermetabolic state makes the liver more susceptible to injury, and, in addition, thyroid hormones might also have a direct toxic effect on hepatic tissue [47]. In almost all the reported cases, the relation of the intrahepatic cholestasis to hyperthyroidism was documented when the jaundice has resolved with hyperthyroidism treatment, and after excluding all other possible causes of cholestasis [45–47]. Histologically, there are mild lobular inflammatory cellular infiltrates in addition to centrilobular intrahepatic cholestasis [46]. In a case series analysis by Fong et al. the liver histology changes due to hyperthyroidism were not characteristic and nonspecific [48]. 

Jaundice due to intrahepatic cholestasis may be a prominent symptom in GD patients, and very occasionally it is the presenting manifestation of thyrotoxicosis [44, 48]. Very high-serum bilirubin levels (up to 581 *μ*mol/L) were occasionally noted in patients with hyperthyroidism [45, 47, 48]. 

The relation of jaundice to GD (or hyperthyroidism in general) can be presented in three clinical scenarios. GD may be the underlying cause of jaundice that develops in a previously healthy subject [47, 49]. The presentation of GD for the first time with jaundice may lead to unnecessary investigations and a delay in management [47]. It is prudent to look carefully for clinical stigmata of thyroid dysfunction, and to consider checking thyroid hormone levels while investigating patients with jaundice of unknown cause. The second clinical scenario develops when a patient with a preexisting chronic liver disease gets deterioration of his liver function tests with deep jaundice. Numerous possibilities are usually considered in this situation including a complicating hepatocellular carcinoma, viral reactivation or superinfection, sepsis, and drug side effects. In this setting, hyperthyroidism should not be omitted as a possible cause. Hegazi et al. reported a case of deep jaundice caused by hyperthyroidism due to a toxic adenoma in a patient with hepatitis B cirrhosis, with return of serum bilirubin to baseline level after treatment with radio-iodine [45]. Thompson et al. reported a patient with primary biliary cirrhosis who had dramatic deterioration of liver functions with jaundice due to the development of GD [50]. The patient's jaundice entirely reversed with treatment of the hyperthyroidism [50]. Thirdly, when a GD patient develops jaundice, a list of possible causes should be considered. These include, an unrelated biliary or hepatic disease [48, 51], an autoimmune liver disease that is known to be associated with GD [46], hepatic congestion due to concomitant congestive cardiac failure [48], hepatic manifestations of hyperthyroidism [47, 49], and hepatotoxic side effects of antithyroid drugs [52]. In the analysis made by Fong et al., severe liver test abnormalities, including deep jaundice occurred in patients with hyperthyroidism alone and with hyperthyroidism with congestive cardiac failure [48]. Drug-induced hepatotoxicity should be considered in those who present with hepatic dysfunction after initiation of thionamide therapy [46, 53]. 

Treatment of a hyperthyroid patient with jaundice needs to be considered and therefore, it will be discussed here. Review of the literature showed that treatment options other than thionamide drugs might have been preferably used in cases of jaundice and hyperthyroidism. In many of the cases the mode of antithyroid therapy was radio-iodine [45, 54], or thyroidectomy [51, 55]. Antithyroid drugs have hepatotoxic side effects in 0.5% of cases with methimazole and carbimazole mainly producing cholestasis, and propylthiouracil mainly causing hepatocellular damage [52]. These side effects are idiosyncratic rather than dose related [46]. Methimazole therapy may deteriorate a GD-related cholestatic jaundice [53]. However, it has been reported that carbimazole and methimazole were successfully used in restoring euthyroidism as well as ameliorating the hyperthyroidism-related jaundice [47, 56]. 

In the absence of another evidence of liver disease, and when jaundice is purely due to the hyperthyroidism, thionamide drugs may be used with monitoring of serum bilirubin and liver function tests. In patients with acute or chronic liver disease who develop GD that aggravates their jaundice, the small probability of hepatotoxic side effects of thionamide drugs may carry the risk of inducing fulminating hepatic failure [51], so that alternative GD treatment options are preferred.

## 5. Right Heart Failure

Thyroid hormone effects on the cardiovascular system include increased resting heart rate, left ventricular contractility, blood volume, and decreased systemic vascular resistance [57, 58]. Cardiac contractility is enhanced and cardiac output may be increased by 50% to-300% over that of normal subjects [57, 58]. The well-recognized cardiovascular manifestations of hyperthyroidism include palpitations, tachycardia, exercise intolerance, dyspnea on exertion, widened pulse pressure, and atrial fibrillation [57, 58]. In spite of the increased cardiac output and contractility, the left ventricular failure that may occur in severe and chronic cases of hyperthyroidism could be explained by a tachycardia-related left ventricular dysfunction, and/or a thyrotoxic cardiomyopathy [57, 58]. The higher prevalence of hyperthyroid heart failure in older age groups signifies the contribution of other cardiovascular comorbidities that include hypertension and coronary artery disease [57]. 

In addition to the well-known presentations, a variety of unusual cardiovascular manifestations are increasingly being reported in association with hyperthyroidism. These include pulmonary arterial hypertension (PH) [59, 60], right heart failure [61, 62], myocardial infarction [63], and heart block [64]. Clinically, isolated right-sided heart failure may be the presenting feature of GD. 

In an echocardiographic study by Marvisi et al., mild PH was found in 43% of the 114 hyperthyroid patients and in none of the healthy control group [59]. In another study by Mercé et al., there was a high prevalence of PH in hyperthyroid patients [60]. Additional studies [65], case series [66], and case reports [61] have shown similar findings. The pathophysiologic link between thyroid disease and PH remains unclear [67]. Possible explanations include immune-mediated endothelial damage or dysfunction, increased cardiac output resulting in endothelial injury, and increased metabolism of intrinsic pulmonary vasodilator substances [60]. Review of the literature reveals some support for the immune-mediated mechanism [68, 69]. In a review by Biondi and kahaly, PH was more linked to GD than to other causes of hyperthyroidism [68]; and in a study by Chu et al., there was a high prevalence of autoimmune thyroid disease in patients with PH [69]. However, in a study by Armigliato et al., the immune mechanism has been questioned because 52% of hyperthyroid subjects with PH did not have evidence of autoimmune thyroid disease [70]. Also in the study by Mercé et al., pulmonary hypertension did not correlate with the cause of hyperthyroidism [60]. Furthermore, Marvisi et al. found no statistical difference in thyroid antibody levels between the hyperthyroid study group and the euthyroid control group and stated that PH could be due to a direct influence of thyroid hormones on pulmonary vasculature [59]. We tend to believe that an effect of excess thyroid hormones may be responsible for the development of PH, especially with the finding of PH also in patients with hyperthyroid nodular goiter.

In spite of the observation that PH was mild in most of the studied hyperthyroid patients [58], cases of severe PH leading to right-sided heart failure are increasingly being recognized [71]. GD occasionally presents with frank isolated right heart failure due to the severe PH [61, 72, 73]. All other possible causes of right ventricular failure including left-sided systolic and/or diastolic dysfunctions have been excluded in reported cases [61]. PH as well as right heart failure showed improvement after the treatment of the concomitant hyperthyroidism [58, 61, 71, 73]. It may take several months for the pulmonary artery pressure to normalize following the initiation of antithyroid treatment [61, 66]. In one case report, the severe pulmonary hypertension has dropped to a near-normal value, only after 14 months from initiation of carbimazole therapy, in spite of a long period of clinical and biochemical euthyroidism [61].

## 6. Conclusions and Recommendations

The unusual manifestations of GD are diverse and affect various body systems. They include hematological, cardiovascular, gastrointestinal, hepatic, and dermatological manifestations (Table 1). Reports of other less frequent or rare presentations like venous thromboembolism [74] and cerebral vasculitis [75] may need further support and documentation. One or more of the unusual manifestations may be the main presenting feature of GD. Awareness about the relation of these presentations to GD or hyperthyroidism is essential to avoid wrong diagnosis and unnecessary investigations. 

The mechanism remains uncertain in the majority of the atypical manifestations. However, a good response to hyperthyroidism treatment is almost guaranteed. The response to hyperthyroidism treatment is either rapid or quite delayed. In the case of vomiting the response occurs within days, however, in the case of right heart failure the improvement occurs within several months from starting the treatment. The excellent recovery that occurs in response to the restoration of euthyroidism makes the effect of excess thyroid hormones the likely underlying mechanism in most of the cases. With the exception of the autoimmune conditions that are associated with GD, the occurrence of the atypical manifestations also in patients with hyperthyroid nodular goiter stands against an autoimmune basis of pathogenesis. 

Such atypical presentations appear to affect significant percentages of GD patients; however, most of the studies conducted in this respect were small. For instance, vomiting was a symptom in 44% of 25 thyrotoxic patients [31], and alkaline phosphatase was raised in 44% of 43 hyperthyroid patients [44]. Larger studies to further evaluate the prevalence of each of the atypical features in GD patients are needed to confirm that some of these findings are not unusual, but are rather under-recognized. The widespread hyperthyroidism manifestations that influence all body systems make us believe that the thyroid hormone effects on various body tissues are not yet fully unveiled.

## Figures and Tables

**Table 1:** Manifestations of Graves' disease (GD).

Well recognized/common	Recognized/Less common	Unusual/atypical (estimated prevalence in GD patients)
Weight loss	Agitation/psychosis	Jaundice (mild hyperbilirubinemia in up to 30%)
Anxiety/nervousness	Apathy/depression	Vomiting (up to 44%)
Tremors	Confusion/delirium	Anemia (up to 33%)
Goiter	Myopathy	Pancytopenia
Tachyarrhythmia	Paraparesis or quadriparesis	Leukopenia/thrombocytopenia
Breathlessness	Abnormal liver function tests	Heart block
Left ventricular failure		Myocardial infarction
Increased bowel movements		Pulmonary hypertension (up to 43%)
Sweating		Right heart failure
Heat intolerance		Angioedema
Staring gaze/exophthalmos		Erythema annulare centrifugum

**Table 2:** Types of Anemia Associated with Graves' disease (GD).

	MCV^¥^	Iron status^#^	Prevalence in GD patients	Response to GD treatment
GD Anemia	Low or normal	Normal or high	22%	Y
Pernicious Anemia	High	Normal	1.4%	N
Iron deficiency Anemia of Celiac Disease	Low	Low	0.9%	N
Autoimmune Hemolytic Anemia	Normal or high	Normal	Only single case reports	Y*

Y: Yes; N: No, **^¥^**mean corpuscular volume, ^#^serum iron, serum ferritin, ±bone marrow iron stores, *may respond to thionamide drug therapy alone.

**Table 3:** Comparison between Graves' disease hyperthyroidism (GD) and Transient hyperthyroidism of hyperemesis Gravidarum (THHG).

	GD	THHG
Hyperthyroidism symptoms^1^	Y	N
Ophthalmopathy	Y	N
Goiter	Y^2^	N^3^
Significant weight loss	Y	N^4^
Severe vomiting	N^5^	Y
TSH	Low (usually <0.01 mU/L)	Low (usually not <0.01 mU/L)
free T4	High (significant rise)	Normal (or mild rise)
Free T3	High	Normal
Persistence >1st trimester	Y	N
Treatment required	Y	N

Y: Yes; N: No, ^1^tremors, marked tachycardia, muscle weakness. ^2^especially with a bruit. ^3^Thyroid gland may enlarge during normal pregnancy. ^4^may be 5% or more in severe cases of HG. ^5^Rarely severe vomiting is a hyperthyroidism feature.
